# Attitudes to antipsychotic drugs and their side effects: a comparison between general practitioners and the general population

**DOI:** 10.1186/1471-244X-6-42

**Published:** 2006-10-18

**Authors:** Josef Helbling, Vladeta Ajdacic-Gross, Christoph Lauber, Ruth Weyermann, Tom Burns, Wulf Rössler

**Affiliations:** 1 Research Unit for Clinical and Social Psychiatry, Psychiatric University Hospital, Zurich, Switzerland; 2 Department of Psychiatry, University of Oxford, Oxford, UK

## Abstract

**Background:**

Attitudes towards antipsychotic medication play an important part in the treatment for schizophrenia and related disorders. We aimed measuring general practitioners' attitudes to antipsychotic drugs and their adverse side effects and comparing these with the attitudes of the general population.

**Methods:**

Analysis and comparison of two representative samples, one comprising 100 General Practitioners (GPs), the other 791 individuals randomly selected from the general population. The setting was the German speaking cantons of Switzerland.

**Results:**

General practitioners have significantly more positive attitudes towards anti-psychotic drugs than the general public. They reject widespread prejudices about the use of anti-psychotic medication significantly more than the general population. In particular the risk of dependency was assessed as 'low' by GP's (80%), in contrast to only 18% of the general population sample. In no instance did a majority of the GPs advise not tolerating any of the 10 possible adverse effects presented in this study. This is in marked contrast to the general population sample, where a majority recommended discontinuation for movement disorder (63%), strong tremor (59%), risk of dependency (55%) and feelings of unrest (54%).

**Conclusion:**

As well as effective management of side-effects being a vital aspect of patient and carer education, prescribing doctors need to be aware that their mentally ill patients are likely to be confronted with extremely negative public attitudes towards antipsychotic medication and with strong pressures to stop taking their medication in the event of side-effects.

## Background

Attitudes towards antipsychotic medication play an important part in the treatment for schizophrenia and related disorders. The effectiveness of antipsychotic medication is evident in acute and maintenance treatment of these disorders, and most mental health professionals recognize antipsychotic drugs as a cornerstone in treating affected people [1]. The general public, on the other hand, are often sceptical and negative in their attitudes to anti-psychotic drugs. They believe the risks of such drugs outweigh the possible benefits [2], and they are negatively stereotyped as being only "symptom alleviating", only "masking the actual problems"[3]. Negative attitudes of people with mental illness towards antipsychotic treatment are known to have a negative impact on their treatment adherence [4-6].

Side effects of antipsychotic drugs such as extrapyramidal symptoms contribute to their poor public perception [3], and there is extensive evidence that distress from side effects is an important contribution to noncompliance [7]. Tolerance of adverse side effects by patients is influenced, among others, by a good doctor patient relationship [8,9], the attitudes of prescribing doctors, and their ability to explain the proposed treatment in an understandable way and to address patients' concerns [10].

Most General Practitioners (GPs) give medical care to psychotic patients. GPs ought to know the attitudes of these patients and should able to make allowances for the divergent perspectives. The goal of this study was to assess GPs attitudes towards anti-psychotic medications and their adverse side effects, and to what extent medical and public opinion on these subjects diverge.

## Methods

This study design compared attitudes towards antipsychotic drugs and their side effects of general practitioners' and the general public using computer assisted telephone (CATI) interviews.

### Samples

#### GPs

A letter outlining the study was sent to 190 general practitioners, selected at random from all GPs in the German speaking part of Switzerland in 2000. The sample was stratified by gender (50%:50%) in order to achieve comparability with the general population sample which had been investigated in a preliminary study [11]. One week after the letter, attempts were made to reach all 190 GPs by telephone. 23 could not be reached having moved, given up their practices, or were long term absent. Of the remaining 167, 100 agreed to participate, resulting in a response rate of 59.8%.

#### General population

The lay sample was derived from a public opinion survey on attitudes towards the mentally ill carried out in 1999. It is a representative sample of the Swiss residential population aged 16 to 76 years (N = 1737). Details about the public opinion survey have been published elsewhere [11,12]. In this analysis, only the interviews from the German speaking part of Switzerland (N = 791) were used. The methodology of the public opinion survey (CATI, questions about antipsychotic medication and tolerance of side effects) was replicated in the present study.

### Variables examined

Firstly, general attitudes towards anti-psychotic drugs were assessed by asking for agreement or disagreement with 7 statements [2] about their effectiveness, benefits and risks and about some widespread prejudices against them (specific questions see table 1). The interviewees were asked to rate each statement on a five point scale from 1 "completely correct" to 5 "completely wrong".

**Table 1:** Percentage distribution of GP's (N = 100) and public attitudes (N = 791) towards statements about antipsychotic medication

	General Practitioners					lay persons					P
			
	disagree	rather disagree	undecided	rather agree	agree	disagree	rather disagree	undecided	rather agree	agree	
1. Antipsychotic drug treatment is the most effective way to treat mental illness	7.1	4	33.3	29.3	26.3	9	17.7	37.1	26.5	9.7	***
2. Antipsychotic drug treatment carries a high risk of dependency	45	35	12	3	5	3.7	14	27.8	33.6	20.9	***
3. The benefits of antipsychotic drug treatment far outweighs the risk associated with it	1	0	13	36	50	2.9	12	40.1	32.9	12.1	***
4. Treatment with antipsychotic drugs can only calm patients down	68.7	19.2	6	5.1	1	15.2	20	28.2	24.8	11.7	***
5. In the long run antipsychotic drugs make one even more ill than before	69	23	7	0	1	15	29.7	29.3	17.2	8.8	***
6. Mentally ill people are only tolerable for their relatives due to antipsychotic drug treatment	3	1	17.2	40.4	38.4	2.4	9	29.3	42.8	16.5	***
7. Since the introduction of antipsychotic drugs, the duration of stay in psychiatric hospitals has become much shorter	0	0	0	24.2	75.8	2.6	9.9	22.7	45.6	19.2	***

*** = p < 0.001 approximate significance level

Secondly, a vignette describing a person who met the DSM-III-R diagnostic criteria for schizophrenia was presented to all interviewees in the GP's sample and to 398 out of the 791 general population sample. Subjects were asked how long the person depicted should be willing to tolerate some side effects of medication (mouth dryness, sweating, tiredness, sexual dysfunction, feeling of unrest, weight gain, visible movement disorders, anhedonia, risk of drug addiction, tremor). The answers were represented by three response categories (never, up to 2–3 weeks, for longer periods). For more details about the questionnaire, see Lauber et al. [12].

### Data analysis

The Mann-Whitney U-test was used to detect differences between the two samples. We performed no correction for multiple testing. The data were computed using the computer package SPSS, Version 9.0 for Windows.

## Results

### General attitudes towards antipsychotic medication

GPs reported generally very positive attitudes towards antipsychotic drugs. A vast majority (86%) thought that the benefits of antipsychotic drug treatment far outweigh the risk associated with it.

All GP's agreed with the assertion that the duration of stay in psychiatric hospitals has shortened since the introduction of antipsychotic drugs, and 79% state, that people with mental illness can only be supported by their relatives due to these treatments. However only 56% agreed with the assertion that antipsychotic drug treatment is the most effective way to treat psychotic illness.

All comparisons between GPs' and the general population's assessments show significant differences, most noticeably on whether antipsychotic drug treatment carries a high risk of dependency. Here 80% of the GPs assessed the risk as low but only 18% of the general population shared this opinion. Also, generalised prejudices against the medication were judged incorrect by the vast majority of general practitioners, whereas only a minority of the general population sample rejected them (Drugs can only calm down patients 88% vs. 35%, and make them even more ill 92% vs. 45%. For complete results see Table 1). On questions about the usefulness of anti-psychotic medication treatment (1, 3, 6 and 7), doctors assessed positive aspects significantly higher.

### Tolerance of side effects

GPs show themselves to be more ambivalent on questions about how long certain antipsychotic drug side effects of should be tolerated (see Table 2). Long-term tolerance was advised despite dry-mouth (83%), heavy sweating (59%), significant weight gain (50%) and risk of drug dependency (68%). In eight out of 10 side effects, more than one third of the general practitioners opted to continue treatment for a further two to three weeks. Advice not to tolerate a side effect at all was never given by a majority of the GPs.

This is in marked contrast to the general population sample. Here a majority voted not to tolerate treatment in the presence of movement disorder (63%), marked tremor (59%), risk of dependency (55%) and feeling of unrest (54%). GPs voted significantly more often for long-term tolerance than did the general population concerning the occurrence of all side effects with the exception of 'continuous feeling of unrest'. In spite of these differences, general practitioners and the public show some similar patterns: Both groups were least accepting of apparent movement disorders, continuing feeling of unrest, marked tremor and persisting anhedonia. Accordingly, the Spearman's r for "no tolerance" of side effects was .88 (p < .001) between GPs and the general population.

## Discussion

The attitudes of mental health professionals and those of the public towards antipsychotic medication may be assumed to diverge. In addition to any personal reservations they may have about their treatment patients taking antipsychotic drugs are under pressure from public attitudes to doubt their benefits and efficacy. It is crucial for successful therapy that patients are well-informed by the prescribing physician and that they are also given comprehensive and appropriate information. Traditionally this is assumed to come from psychiatrists but Kendrick et al. [13] showed that long-term psychotic patients have frequent contact with their GPs and are influenced by their opinions. This study investigated the attitudes of general practitioners towards antipsychotic medication and compared them with the attitudes of the public.

### Principal findings

General practitioners have significantly more positive attitudes towards the use of anti-psychotic drugs and opinions of their effectiveness than do the general public. They reject widespread prejudice about the use of such drugs significantly more strongly than the general public. It is highly likely that they will advise their patients to tolerate possible adverse side effects of these medications longer than lay persons do. There is no published literature on the attitudes of psychiatrists in this area so it is difficult to know if patients are exposed to differing professional opinions.

This study showed that the general population and the GPs have contrary ideas on the risk of drug dependency and whether or not drug dependency should be tolerated, with 80% of the GPs not agreeing that antipsychotics have a risk of dependency whereas the majority of the general population does. The two groups have comparable opinions on how long patients should accept visible movement disorders. Moreover, they both believe that the duration of inpatient stay has become much shorter since the introduction of antipsychotics, but reject the belief that people with mental illness are only tolerable for their relatives due to antipsychotic drug treatment.

### Strengths and weaknesses of the study

This is the first study to assess GPs' attitudes towards antipsychotic drugs, and to compare these results with attitudes in a representative sample of the general population. There is likely to be a degree of bias in our findings as our subjects are likely to be drawn from amongst the more communicative and cooperative members of these populations., Respondents' answers could also be biased by social desirability. However we attempted to minimise this influence by choosing telephone interviews, which are generally considered superior to face-to-face interviews in terms of confidentiality and social desirability. [14].

### Implications for clinicians and policymakers

Patients often doubt the benefits and the efficacy of antipsychotic drugs. They are also less tolerant of adverse side effects than their GPs would consider acceptable for the therapeutic benefits. GPs and the public differ very much in their opinions as to *how long *side effects should be tolerated. The ability to anticipate, manage and tolerate these side effects is a vital component of patient and carer education [8]. Prescribing doctors need also to recognise that their mentally ill patients will be exposed to extremely negative public attitudes towards antipsychotic medication. This will often be accompanied by advice to stop them in the event of side effects. Patients report a general lack of information on the reasons for prescribing anti-psychotics and on the side effects that may accompany them [15,16]. Being better informed, particularly about side effects, should strengthen their resistance to ill-informed public opinion and prejudice.

New developments in psychiatric care such as "shared decision making" [17,18] and an improved assessment and management of side effects [19] need to be more effectively communicated to the public, to improve general understanding and, consequently, attitudes to their use. There is a pressing need for public awareness programmes to raise understanding in this area.

The GPs' attitudes uncovered in this study also call for action. Their willingness to tolerate side effects for two to three weeks and their certain lack of understanding that antipsychotic drugs do not create dependency underline the need to develop education programmes specifically aimed at general practitioners about the rapidly developing field of antipsychotic medication.

## Conclusion

In the context of mental disorders, the premisses regarding the use of antipsychotic drugs differ broadly. Prescribing doctors need to be aware that their mentally ill patients are likely to be confronted with extremely negative public attitudes towards antipsychotic medication. Doctors should anticipate the most relevant prejudices and address them explicitely.

## Competing interests

The author(s) declare that they have no competing interests.

## Authors' contributions

JH carried out the interviews, performed the statistical analysis and drafted the manuscript. VAG assisted in statistical analysis and in writing. CL helped to design the study, coordinate the data bases and helped to draft the manuscript. RW assisted in interviewing and helped to draft the manuscript. TB and WR were involved in interpretating the data and revising the manuscript. All authors read and approved the final manuscript.

**Table 2:** Percentage distribution of GPs' (N = 100) and lay people's opinions (N = 398) as to how long the side effects of antipsychotic drugs should be tolerated

	General Practitioners			lay persons			P
			
	No tolerance	2–3 weeks	Long-term	No tolerance	2–3 weeks	Long-term	
Unpleasant dry mouth	2	15.2	82.8	18.3	49.5	32.2	***
Heavy sweating	3	38.4	58.6	26.1	50.1	23.8	***
Continuous tiredness	5.1	54.5	40.4	24.4	56.9	18.7	***
Frequent sexual dysfunction	4.1	52.6	43.3	27.1	51	21.9	***
Continuous feeling of unrest	43.4	45.5	11.1	53.5	38.3	8.2	n.s.
Significant weight gain	9.3	41.2	49.5	35.9	46.4	17.7	***
Visible movement disorder	48.5	40.4	11.1	62.8	27.8	9.4	*
Continuous anhedonia	24.5	60.2	15.3	33.9	55.4	10.7	*
Risk of drug dependency	22.7	9.1	68.2	55.2	30.1	14.7	***
Marked tremor	33.7	54.1	12.2	58.9	33.7	7.4	***

*** = p < 0.001 * = p < 0.05 approximate significance level

## Pre-publication history

The pre-publication history for this paper can be accessed here:


